# Invited Editorial for Physiological Reports (PHY2‐2020‐04‐0157.R2) “Physiological variations of blood pressure according to gender and age among healthy young black Africans aged between 18 to 30 years in Côte d’Ivoire, West Africa”

**DOI:** 10.14814/phy2.14616

**Published:** 2020-10-14

**Authors:** Jonathan M. Nizar

**Affiliations:** ^1^ Division of Nephrology and Hypertension Carver College of Medicine Fraternal Order of Eagles Diabetes Research Center University of Iowa Iowa City IA USA

Hypertension is a primary cause of cardiovascular disease and death in Low and Middle‐Income Countries which account for two‐thirds of this global health burden (Perkovic et al., 2007). A combination of genetic and environmental factors has primed Africans for increased hypertension prevalence during an accelerated epidemiological transition toward urbanization over the last few decades (Vijver et al., 2013). To anticipate and respond to this epidemic, epidemiologic and physiologic data on hypertension in Africa are needed.

While still comparatively limited, the more abundant data from people of self‐identified African descent living in Europe and North America are not suitable for extension to African communities as it is confounded by variables known to cause profound and generational effects on blood pressure (e.g., mineral and nutritional intake, obesity, access, and utilization of healthcare, etc.). Survey data from several international studies, including WHO STEPS and INTERSALT, have captured the wide range of blood pressures across and within Westernized and African countries (Cooper et al., 2005; Rose & Stamler, 1989), highlighting the significant heterogeneity across populations along multiple dimensions and the danger of generalizing across them. Diversity of severity, prevalence, and risk‐factor associations also exists across the continent and individual African countries as demonstrated by the H3Africa AWI‐Gen study (Gómez‐Olivé et al., 2017). While the study of hypertension pathophysiology may be generalized across epidemiological groups, local survey data are critical to inform robust public health initiatives.

In this issue, Balayssac‐Siransy et al report blood pressure and demographic characteristics of young, healthy black Africans from Cote d’Ivoire. Researchers within Cote d’Ivoire have published only a handful of reports on hypertension prevalence and its associated risk factors (Euloge et al., 2019; Sackou et al., 2019), and none in this age group.

Genetic and environmental effects critically contribute to regional differences in blood pressure and their discovery alters the treatment of hypertension. The RG563Q mutation in beta ENaC, found in 20% of the indigenous San people in South Africa is also found in 5% of hypertensive black South Africans (Jones et al., 2012), but it absent in West African peoples. A possible founding effect generated by the enslavement and transport of Africans to North America may contribute to the lower renin, increased sodium‐sensitivity, and differing drug efficacies observed in black compared to white Americans. In one study, self‐identified African‐Americans were more likely to have non‐dipping diurnal variation and larger left ventricular mass compared to Sub‐Saharan Africans (Fumo et al., 1992). Reports of the blood pressure demographics of Ivorians are a critical first step toward understanding the hypertension epidemic in that country.

Demographic information on clinically‐actionable blood pressure phenotypes, such as resistant hypertension, in Africa are understudied (Nansseu et al., 2016) and due to the continent's rapid epidemiological transition, the prevalence of hypertension is predicted to increase (Vijver et al., 2013). In these contexts, the authors’ report is a meaningful contribution to public health and an important step toward refining our understanding of blood pressure in humans.

## CONFLICT OF INTEREST

The author has no conflicts of interest to disclose.
